# Dietary calories and lipids synergistically shape adipose tissue cellularity during postnatal growth

**DOI:** 10.1016/j.molmet.2019.03.012

**Published:** 2019-04-05

**Authors:** Irina Meln, Gretchen Wolff, Thomas Gajek, Johanna Koddebusch, Sarah Lerch, Liza Harbrecht, Wujun Hong, Irem Bayindir-Buchhalter, Damir Krunic, Hellmut G. Augustin, Alexandros Vegiopoulos

**Affiliations:** 1 DKFZ Junior Group Metabolism and Stem Cell Plasticity, German Cancer Research Center, Im Neuenheimer Feld 280, Heidelberg 69120, Germany; 2 Light Microscopy Facility, German Cancer Research Center, Heidelberg 69120, Germany; 3 European Center for Angioscience (ECAS), Medical Faculty Mannheim, Heidelberg University, Mannheim 67167, Germany; 4 Division of Vascular Oncology and Metastasis, German Cancer Research Center (DKFZ-ZMBH Alliance), Heidelberg 69120, Germany; 5 German Cancer Consortium, 69120, Heidelberg, Germany

**Keywords:** Adipose tissue, Childhood obesity, Nutrient sensing, Progenitor cell proliferation, Programming of disease, Metabolic syndrome

## Abstract

**Objective:**

The susceptibility to abdominal obesity and the metabolic syndrome is determined to a substantial extent during childhood and adolescence, when key adipose tissue characteristics are established. Although the general impact of postnatal nutrition is well known, it is not clear how specific dietary components drive adipose tissue growth and how this relates to the risk of metabolic dysfunction in adulthood.

**Methods:**

Adipose tissue growth including cell proliferation was analyzed in juvenile mice upon dietary manipulation with in vivo nucleotide labeling. The proliferative response of progenitors to specific fatty acids was assayed in primary cultures. Long-term metabolic consequences were assessed through transient dietary manipulation post-weaning with a second obesogenic challenge in adulthood.

**Results:**

Dietary lipids stimulated adipose tissue progenitor cell proliferation in juvenile mice independently of excess caloric intake and calorie-dependent adipocyte hypertrophy. Excess calories increased mitogenic IGF-1 levels systemically, whereas palmitoleic acid was able to enhance the sensitivity of progenitors to IGF-1, resulting in synergistic stimulation of proliferation. Early transient consumption of excess lipids promoted hyperplastic adipose tissue expansion in response to a second dietary challenge in adulthood and this correlated with abdominal obesity and hyperinsulinemia.

**Conclusions:**

Dietary lipids and calories differentially and synergistically drive adipose tissue proliferative growth and the programming of the metabolic syndrome in childhood.

## Highlights

• Dietary fat accelerates adipose tissue progenitor proliferation in juvenile mice.
• Lipid-mediated proliferation is independent of excess calorie intake.
• Excess calories elevate IGF-1 levels and adipocyte hypertrophy.
• Palmitoleic acid enhances the proliferative response of progenitors to IGF-1.
• Lipids and calories in childhood program features of the adult metabolic syndrome.

## 1 Introduction

The quantity, anatomical distribution, and functionality of adipose tissue as a lipid storing and endocrine organ are defining features and key drivers of the metabolic syndrome [1], [2], [3], [4]. Obesity and abdominal visceral adiposity in particular are major risk factors for type 2 diabetes and cardiovascular disorders. Accumulating evidence suggests that childhood and adolescence are critical periods for shaping adipose tissue properties and thereby determining the risk of obesity and metabolic disease. Overweight at kindergarten age was shown to increase the risk of later obesity by 4-fold [5], and similar findings were reported for school-age children [6], [7]. In fact, acceleration of BMI or general early growth *per se* can increase later susceptibility to abdominal obesity and the metabolic syndrome [8], [9], [10]. There is little doubt about the role of inadequate nutrition as a driving force for early-onset obesity and its rising prevalence. However, the contribution of excess calorie intake, dietary fat, and other nutritional factors is currently unclear [11], [12], [13]. In particular, it is not known how components of early nutrition promote stable changes in the adipose tissue cellular composition thereby programming long-term tissue function, fat distribution, and systemic metabolism in adulthood.

Systemic nutrient excess promotes lipid storage in adipocytes and consequently adipocyte hypertrophy, resulting in enlargement of the fat depot, a reversible process contributing to obesity in young and adult age [14], [15], [16]. However, both childhood and adult obesity are also characterized by higher quantity of adipocytes (hyperplasia), representing a temporally more stable property [16], [17], [18]. Adipocytes are generated by the differentiation of resident immature adipocyte precursor/progenitor cells, which is stimulated by the consumption of diets with a high fat and calorie content (HFD) in rodents [19], [20], [21], [22], [23]. Furthermore, HFD has been shown to acutely promote progenitor cell proliferation, thereby increasing the potential for hyperplastic growth [19]. However, consumption of excess calories under diets with low fat content can also increase progenitor proliferation and hyperplasia [19], [24].

Thus, the control of progenitor proliferation and hyperplasia by dietary components and the involvement of systemic and local factors mediating nutrient excess are poorly understood. We have investigated the role of excess calories and dietary lipids in adipose tissue proliferative growth in juvenile mice as well as the link between diet-dependent progenitor responses and the susceptibility to abdominal obesity and the metabolic syndrome in adulthood.

## 2 Materials and methods

### 2.1 Mice

Female C57Bl6/N mice were purchased from Charles River Laboratories at 2 weeks of age with dam or at 6 months as indicated. Female B6-Cd36^tm1Mfe^ (Cd36-KO) mice [25] were kindly provided by Dr. Peter Voshol, Leiden University Medical Center. Mice were housed on control diet (CD, D12450Bi, Research Diets, New Brunswick, NJ, USA) for 1 week. At weaning, mice were earmarked and randomized by body weight for feeding with CD, high fat diet (HFD, D12492i, Research Diets, New Brunswick, NJ, USA), or HFD with equal calorie load as CD (restricted high fat diet, R-HFD) for the indicated duration. Equal numbers of mice across groups were assigned to each cage (3–5 mice per cage). The amount of diet consumed by the CD group was measured daily. The amount of HFD equivalent to the calorie load consumed by the CD group was provided to R-HFD cages on a daily basis. NMR quantitation (EchoMRI, Houston, TX, USA) was used to analyze body composition. For EdU administration, mice received EdU (5-ethynyl-2′-deoxyuridine, E10187, Thermo Fisher Scientific, Darmstadt, Germany) dissolved in drinking water at 0.2 mg/ml for the indicated duration. Insulin tolerance test (ITT) was performed by injecting intraperitoneally 1 U insulin (HUMINSULIN, Lilly, Germany) per kg body mass after a 5-h fast. Blood glucose was determined with an Accu-Check glucometer (Roche Diagnostics, Germany). Mice were euthanized by cervical dislocation. Animal procedures were performed in accordance with the European Union directives and the German animal welfare act (Tierschutzgesetz) and approved by local authorities (Regierungspräsidium Karlsruhe).

### 2.2 Flow cytometry including FACS and *in vivo* EdU incorporation analysis

The stromal-vascular fraction (SVF) was obtained from gWAT and ingWAT as described by Bayindir et al. [26]. Erythrocytes were depleted by magnetic separation. Briefly, SVF cells were incubated in CD16/32 FcBlock (14-0161-85, ebioscience, Germany) for 5–10 min on ice following incubation with Ter119-microbeads (130-049-901, Miltenyi Biotec, Germany) for 15 min on ice. Cell suspension was applied on pre-separation filter inserted into magnetic column placed into an OctoMACS Separator (Miltenyi Biotec, Germany). The flow-through was collected and incubated with the following antibodies: CD45-AF700 (30-F11, 56-0451-82, eBioscience, Germany), CD31-APC (390, 17-0311-82, eBioscience, Germany), CD34-eFluor450 (RAM34, 48-0341-82, eBioscience, Germany), CD29-PE (eBioHMb1-1, 12-0291-81, eBioscience, Germany), Sca1-APC-Cy7 (30-F11, 56-0451-82, eBioscience, Germany). Cells were sorted with a BD FACS Aria (BD Biosciences) and directly processed for RNA isolation. For EdU-labeling, the Click-iT™ Plus EdU Alexa Fluor™ 488 Flow Cytometry Assay Kit (C10633, Thermo Fisher Scientific, Germany) was used according to the manufacturer's protocol. Cells were analyzed with BD FACS LSR II (BD Biosciences, Germany) using BD FACSDiva™ software (BD Biosciences, Germany). Gating for EdU was done based on fluorescence minus one (FMO) control, stained with all antibodies and without EdU.

### 2.3 Histological analysis by hematoxylin & eosin staining

gWAT were fixed with 4% Histofix (Roth, Germany) at room temperature for 24 hours then embedded in paraffin. Blocks were cut on RM2245 microtome (Leica, Germany) into sections 5-μm thick each and placed on glass slides. Hematoxylin and eosin (H&E) staining was performed as follows: rehydration following by hematoxylin staining (GHS332-1L, Sigma) for 5 min, 0.4% HCl for 20 seconds, eosin staining (HT110132-1L, Sigma, Germany) for 30–60 seconds and dehydration. For mounting, Eukitt® (03989-100 ML, Sigma) was used. Microscopy was performed using a motorized Cell Observer.Z1 microscope (Zeiss, Jena, Germany) equipped with the AxioCam MRm CCD camera. Adipocyte lipid droplet area was estimated automatically with constant settings using an ImageJ macro (https://imagej.nih.gov/ij/). 5066 events per mouse were quantified on average. Briefly, adipocytes were segmented and area was determined. Adipocyte diameter and volume were estimated based on area using following formulas: diameter = 2√ (mean cell area/π) μm and volume = π (diameter3/6) μm^3^. Number of adipocytes per gram of adipose tissue was calculated as following: 1) conversion of the volume from μm^3^ to a volume in picolitres (pl) (1 pl = 1000 μm^3^), 2) calculation of the mass of a cell, where mass = volume × density and the density of adipose tissue can be assumed to be 0.96 g ml^−1^, mean cell mass = mean cell volume × 0.96, 3) calculation of the number of cells per mg of tissue as number of cells per mg tissue = 1/mean cell mass. The absolute number of adipocytes per depot was estimated as = number of adipocytes per gram × gram mass of depot.

### 2.4 Histological analysis by Caveolin-1 staining including EdU incorporation

gWAT was dissected, fixed, and sectioned as described above. Immunohistochemistry was performed after xylene de-paraffinization and ethanol dilutions to rehydrate. Sections were subjected to antigen retrieval in citrate buffer (pH 6.0) by boiling at 95 °C for 20 min. The slides were blocked with 2% BSA in PBS for 1 hour at room temperature followed by overnight incubation at 4 °C with rabbit α-Caveolin-1 polyclonal antibody, 1:400 in 2% BSA in PBS (#3238, Cell Signaling, Danvers, MA, USA). Following washes with PBS, goat α-rabbit IgG-Alexa Fluor® 488 secondary antibody at 1:400 dilution in 2% BSA/PBS (Thermo Fisher Scientific, Rockford, IL, USA) was applied for 60 min at room temperature. The sections, protected from light, were washed and 0.1% Triton-100 in PBS was applied for 30 min to permeabilize. Slides were washed with PBS and Alexa Fluor™ 594 EdU Imaging Kit (C10639, Thermo Fisher Scientific, Germany) was used, according to manufacturer's instructions, to detect EdU incorporated cells. Following 30 min ClickIT reaction, slides were washed in PBS and Hoescht (1:2000 dilution in PBS) was applied for 30 min, slides were washed finally, and mounted with ProLong® Gold Antifade Reagent (Cell Signaling, Danvers, MA, USA) and coverslips. Images were acquired at 100× magnification using the Zeiss Cell Observer with identical settings and acquisition times for all samples. Images were analyzed with Figi software (ImageJ, https://imagej.nih.gov/ij/). The automatic image segmentation and analysis of DAPI/EdU signal in adipocytes was performed using FIJI (ImageJ, http://rsbweb.nih.gov/ij) macro. 909 adipocytes (EdU staining, Fig. S3) or 187 adipocytes (no EdU staining, Figure 4) were quantified per mouse on average. Shortly, images were background subtracted using Rolling ball algorithm, Median filtered, and Skeletonized. The images were analyzed using Analyze Particles tools and the cell selections were added to the Region of interest (ROI) manager. The DAPI and EdU images were background subtracted, Gaussian blurred, and the Find Maxima tool was used to identify the maxima intensities corresponding to the centers of masses. Finally, the DAPI and EdU maxima intensities were analyzed within adipocytes ROIs.

**Figure 1:**
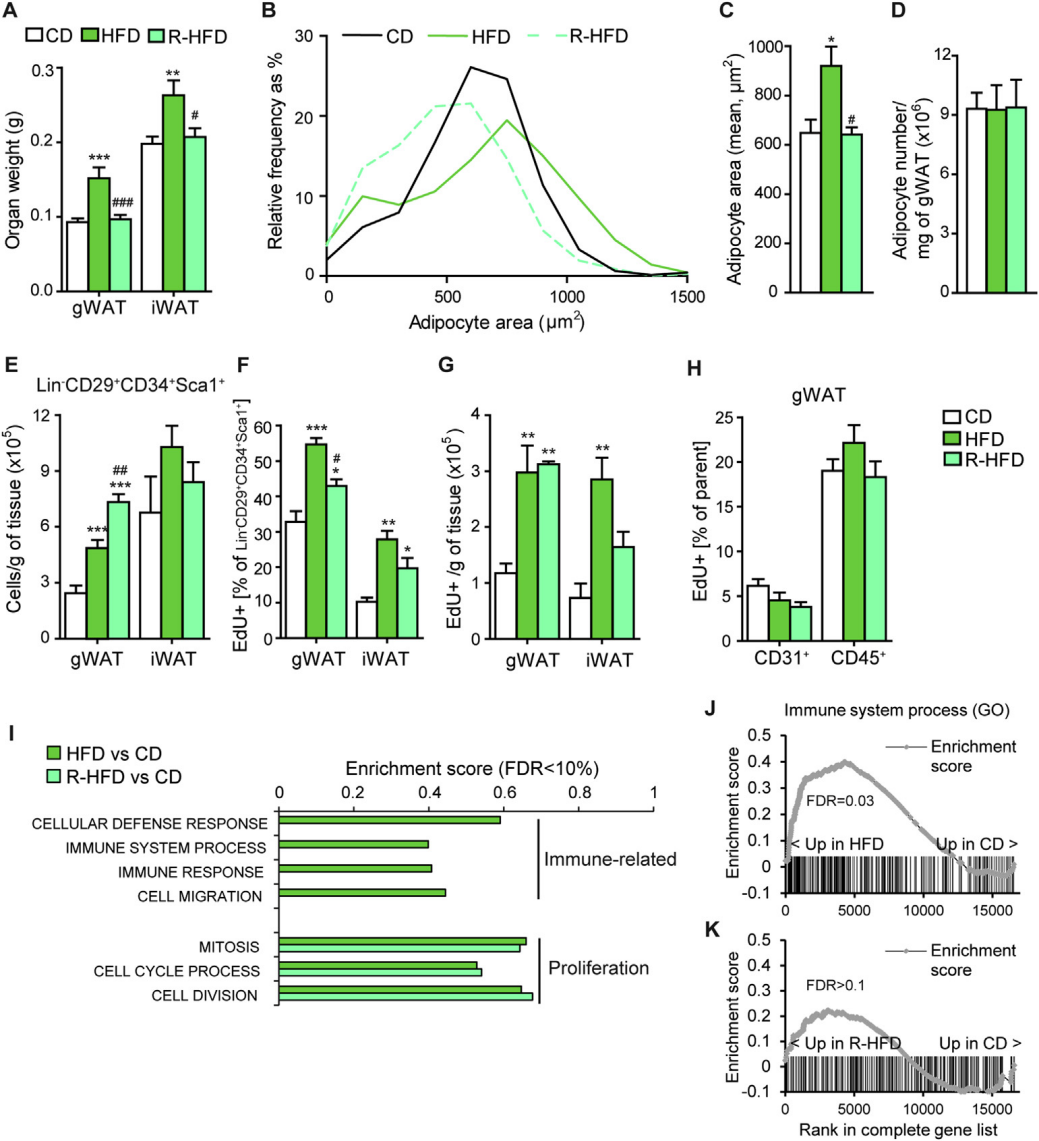
Dietary lipids accelerate adipose tissue progenitor proliferation independently of excess calories and adipocyte hypertrophy in juvenile mice. (A) Weight of gonadal (gWAT) and subcutaneous (iWAT) fat depots of 3-week-old female mice after one week of control diet (CD), high-fat diet (HFD) or HFD calorically matched to CD (R-HFD) (n = 10 mice/group). (B) Relative frequency distribution of adipocyte area in gWAT after one week of CD, HFD, and R-HFD, determined by quantitative microscopy of H&E-stained sections (n = 6 mice/group). (C, D) Mean adipocyte area (C) and number of adipocytes per gram of gWAT (D) after one week of CD, HFD and R-HFD, calculated from data in (B) (n = 6 mice/group). (E) Number of Lin^−^CD29^+^CD34^+^Sca1^+^ cells per gram of tissue after one week of CD, HFD, and R-HFD, determined by flow cytometry (n = 4 samples/group with cells pooled from 3 mice each). (F, G) Frequency (F) and number per gram of tissue (G) of EdU^+^ Lin^−^CD29^+^CD34^+^Sca1^+^ cells after one week of CD, HFD, and R-HFD with EdU-containing drinking water, determined by flow cytometry (n = 4 samples/group with cells pooled from 3 mice each). (H) Frequency of *in vivo* EdU incorporation in CD31^+^ and CD45^+^ cells in gWAT of mice shown in (F), determined by flow cytometry. (n = 4 samples/group with cells pooled from 3 mice each). (I) Representative selection of Gene Ontology (GO) gene sets with False Discovery Rate (FDR) < 10% from Gene Set Enrichment Analysis (GSEA) on expression profiles from gWAT after one week of the indicated diets (n = 3 (CD), 5 (HFD), 5 (R-HFD) samples, each pooled from 2 mice). (J, K) Enrichment plots for the indicated gene sets from GSEA (I). Vertical bars indicate the rank of the individual genes of the indicated gene set in the ranking of the transcriptome by differential expression (n = 3 (CD), 5 (HFD), 5 (R-HFD) samples, each pooled from 2 mice). Data are presented as mean ± SEM. *P < 0.05, **P < 0.01, ***P < 0.001 vs. CD and ^#^*P* < 0.05, ^##^*P* < 0.01, ^###^*P* < 0.001 vs. HFD (One-way ANOVA, posthoc Tukey).

**Figure 2:**
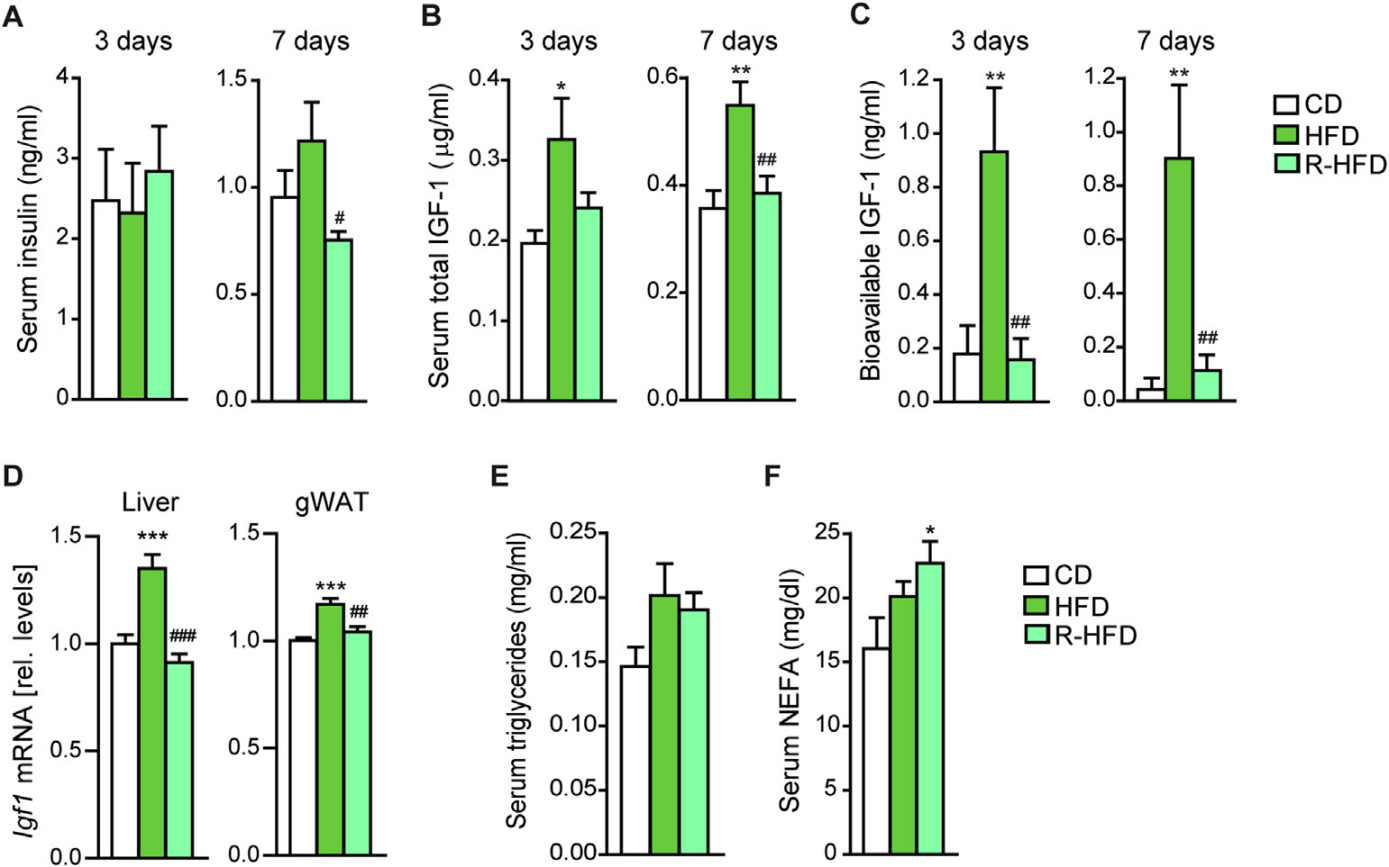
Excess dietary calories, but not lipids, increase circulating IGF-1 levels. (A–C) Serum insulin (A), total IGF-1 (B) and bioavailable IGF-1 (C) after 3 (postprandial) or 7 (*ad libitum*) days of CD, HFD and R-HFD in juvenile mice, determined by ELISA (n = 10 mice/group for each time point). The postprandial state represents 3 hours of refeeding after food withdrawal in the light phase. (D) *Igf1* mRNA expression in liver and gWAT of CD-, HFD-, and R-HFD-fed mice (gWAT n = 5 samples/group, each pooled from 2 mice, liver n = 10 mice/group). (E, F) Serum triglyceride (E) and non-esterified fatty acids (F) after 3 days (postprandial) of CD, HFD, and R-HFD in juvenile mice, determined biochemically (n = 10 mice/group). Data are presented as mean ± SEM. *P < 0.05, **P < 0.01, ***P < 0.001 vs. CD and ^#^*P* < 0.05, ^##^*P* < 0.01, ^###^*P* < 0.001 vs. HFD (One-way ANOVA, posthoc Tukey).

**Figure 3:**
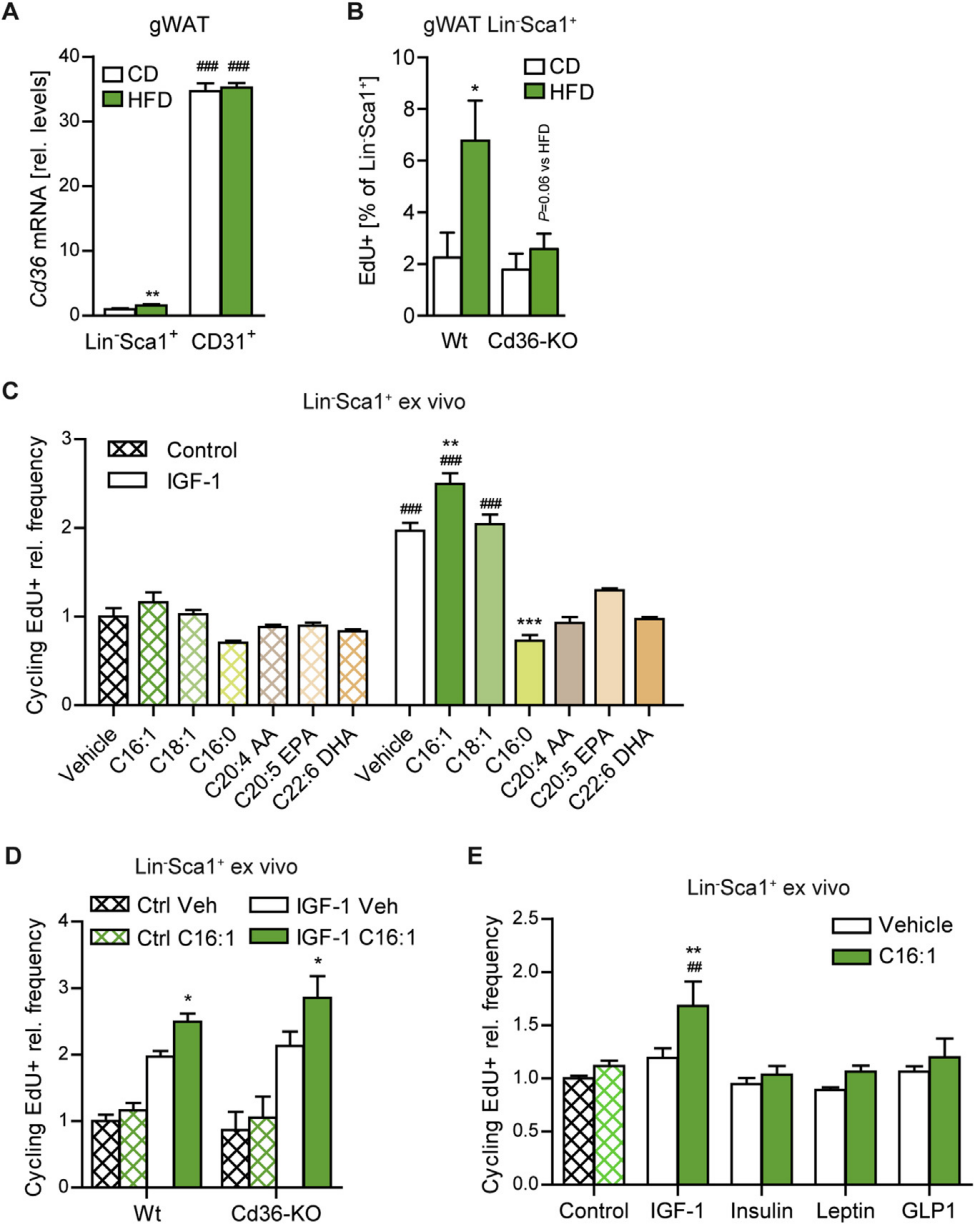
Dietary fatty acids can directly enhance growth factor sensitivity to promote adipose tissue progenitor proliferation. (A) *Cd3*6 mRNA expression in Lin^−^CD29^+^CD34^+^Sca1^+^ or CD31^+^ cells sorted by FACS from gWAT of mice fed CD or HFD for 1 week (n = 4 samples/group, each pooled from 2 to 3 mice). (B) Frequency of EdU^+^ Lin^−^Sca1^+^ cells in wildtype (Wt) or Cd36-knockout mice after one week of CD, HFD, and R-HFD with EdU-containing drinking water, determined by flow cytometry (n = 4 mice/group). (C–E) Relative frequency of EdU^+^ cycling (S/G2/M) Lin^−^Sca1^+^ cells in primary cultures from wild type (C,E)) or Wt/Cd36-KO (D) gWAT following treatment with fatty acids (250 μM C16:1, C18:1, C16:0; 100 μM C20:4, C20:5, C22:6) and IGF-1, insulin (50 ng/ml), leptin (20 ng/ml) or GLP-1 (100 nM) as indicated for 16 hours in the presence of EdU [relative to Vehicle (Veh), n = 3–7 independent biological replicates/group, each pooled from >2 mice, except for C20:4, C20:5 and C22:6 (technical replicates, descriptively)]. C16:1: palmitoleic acid, C18:1: oleic acid, C16:0: palmitic acid, C20:4 AA: arachidonic acid, C20:5 EPA: eicosapentaenoic acid, C22:6 DHA: docosahexaenoic acid. Data are presented as mean ± SEM. *P < 0.05, **P < 0.01, ***P < 0.001 vs. CD (A,B) or Vehicle (C–E) and ^#^*P* < 0.05, ^##^*P* < 0.01, ^###^*P* < 0.001 vs. Lin^−^Sca1^+^ (A) or Control (C,E) [2-way ANOVA, posthoc Tukey (A,B) or Holm-Sidak (C–E)].

**Figure 4:**
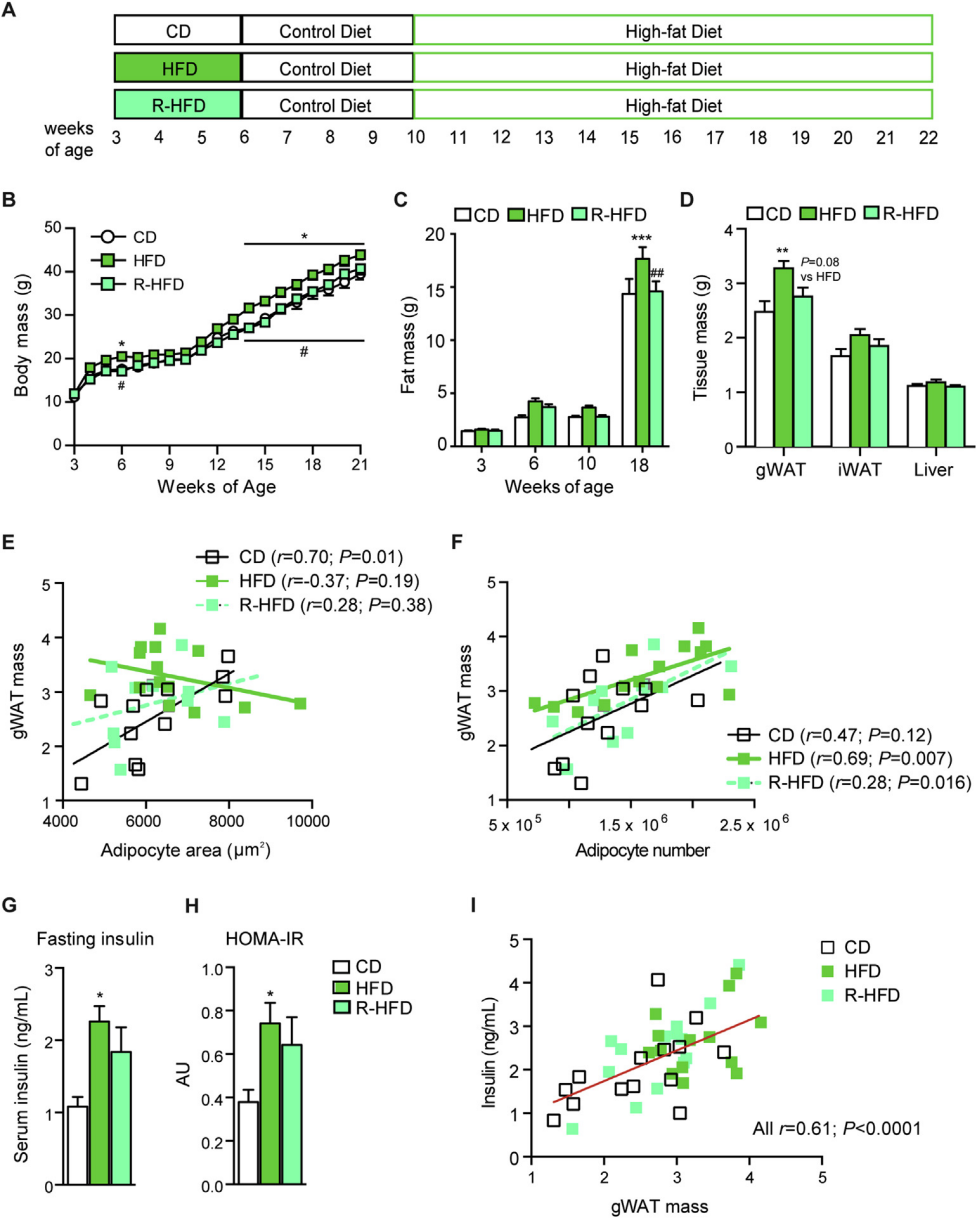
Excess calories and fat during post-weaning growth differentially alter the susceptibility to abdominal hyperplastic obesity and hyperinsulinemia in adulthood. (A) Experimental design for Figure 4. 3-week-old mice were fed CD, HFD, or R-HFD for 3 weeks, followed by control diet for 4 weeks and subsequently HFD for 12 weeks. (B) Body mass (n = 14 mice/group). 2-way repeated measures ANOVA with Tukey posttests. (C) Body fat mass, determined by NMR quantitation (n = 14 mice/group). (D) Organ weights at 22 weeks of age (woa) (n = 14 mice/group). (E, F) Pearson correlation analysis of gWAT mass and mean adipocyte area (E) or total adipocyte number (F), determined by quantitative microscopy of Cav-1-stained sections at 22 woa (n = 12–14 mice/group). (G) Fasting serum insulin, determined by ELISA at 18 woa (n = 6 mice/group). (H) HOMA-IR, calculated from fasting serum insulin and blood glucose concentrations, determined by ELISA and glucometer, respectively, at 18 woa (n = 6/group). (I) Pearson correlation analysis of fasting insulin (ELISA) and gWAT mass at 22 woa (n = 12–14 mice/group). Data are presented as mean ± SEM (B-D, G and H) or as individual mice (E, F and I). *P < 0.05, **P < 0.01, ***P < 0.001 vs. CD and ^#^*P* < 0.05, ^##^*P* < 0.01 vs. HFD (One-way ANOVA with Tukey posttests unless otherwise indicated).

### 2.5 Serum insulin, total IGF-1 and bioavailable IGF-1 ELISA

Concentration of insulin, total IGF-1 and bioavailable IGF-1 in the blood serum was determined by Insulin (Mouse) ELISA Kit (80-INSMS-E01-AL, ALPCO, USA), Insulin-like Growth Factor 1 (Mouse) AssayMax ELISA Kit (EMI1001-1, AssayPro, USA) and Free Rat and Mouse IGF-I ELISA Kit (AL-136, Ansh Labs, USA) respectively according to the manufacturer's protocol. The absorbance was measured on 450 nm using the Mithras Microplate Reader (Berthold Technologies GmbH & Co, Germany). Concentration was calculated based on standard dilution series using Elisa analysis web-resource (http://www.elisaanalysis.com/). HOMA-IR was calculated using the formula: HOMA-IR (mmol l^−1^ × μU ml^−1^) = fasting blood glucose (mmol l^−1^) × fasting serum insulin (μU ml^−1^)/22.5.

### 2.6 Serum non-esterified fatty acid (NEFA) and triglycerides

To determine NEFAs and triglycerides, 2 μl of serum were used in duplicate to measure using the NEFA-HR (2) kit (434-91795, 436-91995, Wako Diagnostics, USA) and the Serum Triglyceride Determination Kit (TR0100, Sigma Aldrich, Germany), respectively. The absorbance was measured on 550 nm using the Mithras Microplate Reader (Berthold Technologies GmbH & Co, Germany).

### 2.7 MACS isolation and culture of adipose tissue progenitors

To isolate progenitor cells, gWAT was dissected and digested as described in Bayindir et al. [26]. Following blocking with CD16/32 FcBlock (14-0161-85, ebioscience, Germany), cells were incubated with CD31-biotin (clone 390, eBioscience, Germany), Ter119-biotin (clone 30-F11, eBioscience, Germany) and CD45-biotin (clone Ter-119, eBioscience, Germany) followed by streptavidin microbeads (Miltenyi Biotec, Germany). Cells were filtered through MACS pre-separation filters with columns (Miltenyi Biotec, Germany) using a magnetic OctoMACS Separator (Miltenyi Biotec, Germany). The flow-through was washed and collected. Lineage depleted cells were then incubated with anti-Sca1 microbeads (clone D7, Miltenyi Biotec, Germany) and collected using a second round of MACS pre-separation filters with columns (Miltenyi Biotec, Germany). The Lin^−^Sca1^+^ fraction cells were counted using a hemocytometer and seeded at 2*10^4^ per cm^2^ in DMEM supplemented with 100 U/ml penicillin-streptomycin (pen-strep, Thermo Fisher Scientific), 10% (v/v) fetal calf serum (FCS, Life Technologies).

### 2.8 Fatty acid cell culture treatments and EdU cell cycle analysis

Fatty acid treatment of Lin^−^Sca1^+^ cultures was performed following 2 days of culture without passaging. Palmitoleic acid (Sigma, P9417), palmitic acid (Sigma, P5585), oleic acid (Sigma, O1008), arachidonic acid (Cay90010-50, Biomol, Germany), eicosapentaenoic acid (Cay90110-50, Biomol) and docosahexaenoic acid (Cay90310-50, Biomol) were dissolved in 100% ethanol to obtain a 200 mM stock solution. Fatty acids were added to treatment medium (DMEM, 0.1% FCS, 0.5% BSA) at the indicated final concentration and the medium was vortexed briefly and incubated in a shaker at 80 rpm at 37 °C for 3 h. Cultures were treated with the fatty acid media for 16 hours in the presence of 5 μM 5-ethynyl-2′-deoxyuridine (EdU, Thermo Fisher Scientific) with or without 50 ng/ml rec. murine IGF-1 (R&D Systems), 50 ng/ml rec. human insulin (Sigma), 100 nM rec. human Glucagon-Like Peptide 1 (GLP-1) Amide Fragment 7-36 (Sigma) or 20 ng/ml rec. mouse leptin (Sigma). EdU incorporation and DNA content analysis was performed with the Click-iT Plus EdU Alexa Fluor 647 Flow Cytometry Assay Kit and FxCycle PI/Rnase staining according to the manufacturer's instructions (Thermo Fisher Scientific). Cells were analyzed with BD FACSCanto II flow cytometer using BD FACSDiva™ software (BD Biosciences). Data analysis was performed using FlowJo software (FlowJo, LLC, USA). Data from independent experiments (proportion of EdU^+^ cells in S/G2/M phase) were mean-scaled within each experiment, averaged and expressed relative to the vehicle/control group.

### 2.9 RNA isolation and microarray expression analysis

After dissection, tissues were frozen in liquid nitrogen and pulverized in Mikro-Dismembrator S (Sartorius, Germany). Tissue powder or sorted cells were lysed with Qiazol (Qiagen, Hilden, Germany). RNA was isolated from tissue lysates using RNeasy Micro Kit (Qiagen, Hilden, Germany) according to the manufacturer's protocol application of the RNase-Free DNase Set (Qiagen, Hilden, Germany) for 15 min. RNA quality control and Microarray expression profiling were performed by Genomics and proteomics core facility (GPCF, DKFZ, Heidelberg) using Affymetrix GeneChip® Mouse Gene 2.0 ST Arrays (Affymetrix, High Wycombe, UK), RMA normalization, visualization and quality controls were done via Affymetrix® Expression Console™ Software. The dataset is available at ArrayExpress (Accession E-MTAB-7755). Differential gene expression was analyzed by LIMMA using TM4: MultiExperiment Viewer (MeV) software [27], [28]. *P* values were adjusted according to Benjamini-Hochberg. Gene set enrichment analysis (GSEA) was performed on the complete probe dataset using the GO gene set collection (Molecular Signatures Database, MSigDB; http://www.broadinstitute.org/gsea/msigdb/index.jsp) [29]. Ranking of gene sets was by the false discovery rate (FDR).

### 2.10 Statistical analysis

Data were graphed and analyzed using GraphPad Prism software (La Jolla, CA, USA). n numbers represent replicates per experimental group (mice, samples). Pearson correlation analysis, two-sided unpaired *t*-test, one-way ANOVA or two-way (repeated-measures) ANOVA with Tukey (*in vivo*) or Holm-Sidak (cell culture experiments) post hoc tests was performed as indicated depending on the experimental design. p < 0.05 was considered statistically significant.

## 3 Results

### 3.1 Dietary lipids accelerate juvenile adipose tissue progenitor proliferation independent of excess calories and adipocyte hypertrophy

In order to determine the differential contribution of high dietary fat and excess calorie consumption to early post-lactation adipose tissue growth, we employed a pair-feeding approach by feeding female mice either a high-fat diet (HFD) *ad libitum* or a HFD load calorically matched (R-HFD) to the consumption of a group of mice on control diet (CD) (Figure S1A). The treatment started at 3 weeks of age, corresponding approximately to school-age and early puberty in humans based on sexual maturity [30]. We focused on the first week of feeding to address direct effects of nutritional components on growth processes. After 7 days of feeding, HFD mice showed significantly higher depot mass in intra-abdominal gonadal (gWAT) and subcutaneous fat (iWAT), which was due to adipocyte hypertrophy with no change in the total adipocyte number, whereas R-HFD mice did not show any fat accumulation or adipocyte hypertrophy (Figure 1A–D). We next quantified the proliferative response of immature precursor and progenitor cells, termed “progenitors” hereafter [19]. Flow cytometry of the stromal-vascular fraction revealed an increased density of Lin^−^CD29^+^CD34^+^Sca1^+^ cells in the gWAT depot upon the 7-day HFD feeding independently of calorie load (Fig. 1E and S1B). This effect was not detectable in the iWAT depot. To determine the rate of cell proliferation we measured the *in vivo* incorporation of 5-ethynyl-2-deoxyuridine (EdU), which was administered over 7 days (Figure S1B). In agreement with previous reports on sexually mature young mice [19], we observed a major increase of progenitor cell proliferation in the gWAT and iWAT depots in HFD-fed mice (Figure 1F,G). Intriguingly, the calorically restricted R-HFD diet was sufficient to substantially increase the rate of cell proliferation in post-weaning mice, resulting in higher density of EdU^+^ progenitors in particular in the gWAT depot (Figure 1F,G). This response was specific to progenitor cells, since HFD feeding did not alter the proliferation rate of CD31^+^ endothelial cells or CD45^+^ leucocytes (Figure 1H). We also profiled the tissue remodeling response of gWAT at the level of gene expression (Figure S1C,D). Consistently, pathways related to cell proliferation predominated the highest ranking gene sets induced by 7 days of HFD compared to CD (Table S1). Interestingly, several gene sets related to immune responses were also upregulated by HFD (Table S1 and Figure 1I,J). Immune-related and inflammatory pathways have been suggested to promote adipose tissue remodeling and progenitor proliferation [31], [32]. However, although R-HFD promoted proliferation-related gene sets to the same extent as HFD, it did not cause a significant enrichment of immune-related pathways (Figure 1I–K, S1E,F and Table S2). Notably, treatment of adult non-growing mice at 6 months of age with HFD/R-HFD resulted in trends of increased cell proliferation which were at overall lower rates compared to juvenile mice and did not increase the number of progenitors in the tissue (Figure S2A–C). Taken together, these findings unexpectedly demonstrate that high intake of dietary fat specifically stimulates adipose tissue progenitor proliferation in juvenile mice independently of excess calorie intake, adipocyte hypertrophy, and obesity.

### 3.2 Excess calories and fatty acids differentially enhance growth factor expression and sensitivity to drive progenitor proliferation

We next interrogated the involvement of mediators of the systemic anabolic state in the promotion of diet-dependent adipocyte progenitor proliferation during post-lactation growth. Hyperinsulinemia is a common feature in obesity, and insulin can promote proliferation in certain cell types [33], [34]. We examined circulating insulin at 3 days of feeding, when a peak of HFD-induced cell proliferation has been shown to occur [19]. However, we could not detect any increase in postprandial serum insulin at 3 days of HFD/R-HFD or in the *ad libitum* fed state at 7 days (Figure 2A). Insulin-like growth factor 1 (IGF-1) represents a critical signal for systemic postnatal growth and can promote preadipocyte proliferation *in vitro* but its regulation by obesogenic nutritional factors remains poorly characterized [35], [36]. HFD feeding resulted in a marked increase in total and bioavailable serum IGF-1 within 3 days but remarkably, this effect was completely absent in R-HFD mice (Figure 2B,C). This is likely to be due to the regulation of *Igf1* mRNA levels in the liver, a major contributor of circulating IGF-1, although HFD also caused a mild but significant increase in *Igf1* expression in adipose tissue (Figure 2D).

The question arose of how circulating lipid levels were linked to systemic IGF-1 levels and the adipose tissue proliferative response under hypercaloric versus isocaloric HFD feeding. At 3 days of feeding, HFD and R-HFD feeding led to a similar trend of increased postprandial serum triglycerides, whereas non-esterified fatty acids where significantly elevated only in R-HFD mice (Figure 2E,F), indicating a dissociation of lipid excess from IGF-1 regulation. To interrogate whether high local supply of non-esterified fatty acids was required for the proliferative response of adipocyte progenitors we examined mice deficient for *Cd36*, encoding a transporter involved in peripheral fatty acid uptake. Cd36-KO mice display increased serum triglycerides and fatty acids but reduced uptake into adipose tissue [25], [37]. In accordance with its role in fatty acid transport across the blood vessel, *Cd36* expression was markedly higher in CD31^+^ endothelial cells compared to progenitor cells (Figure 3A). Remarkably, *Cd36*-KO mice did not increase EdU incorporation in adipose tissue progenitors upon HFD (Figure 3B and S2D), suggesting an essential role of Cd36 in the local proliferative response to systemic lipid excess.

We next used primary cultures of progenitors to address the direct stimulation of cell proliferation by fatty acids and the calorie-dependent IGF-1. Palmitic acid (C16:0) and oleic acid (C18:1), are the most abundant fatty acids in the HFD diet. Although less abundant in the diet, palmitoleic acid (C16:1) and polyunsaturated fatty acids have been shown to have physiologically relevant signaling functions including mitogenic actions [38], [39]. None of the fatty acids tested increased the incorporation of EdU in the absence of IGF-1 (Figure 3C and S2E,F). IGF-1 by itself stimulated the abundance of EdU^+^ cycling cells by 2-fold (Figure 3C). Interestingly, this response was blunted by C16:0 and the polyunsaturated C20:4 (ω-3), C20:5 and C22:6 (ω-6) fatty acids. In contrast, C16:1 was the only lipid with mitogenic activity, enhancing the response to IGF-1 by 25%. This process was independent of Cd36 which is consistent with a role of Cd36 in endothelial transport rather than in progenitors (Figure 3D). Furthermore, we tested the ability of C16:1 to synergize with insulin, leptin or glucagon-like peptide-1 (GLP-1), representing endocrine mediators of the feeding state with the ability to promote cell proliferation [34], [40]. Despite the treatment with relatively high concentrations, the proliferative action of C16:1 was specific to IGF-1 (Figure 3E). In conclusion, these findings indicate that calorie excess could drive adipose tissue progenitor proliferation through elevated IGF-1 levels, whereas specific fatty acids act on progenitors to increase their sensitivity and proliferative response to IGF-1.

### 3.3 Excess dietary calories and fat during post-weaning growth differentially alter the susceptibility to abdominal hyperplastic obesity and hyperinsulinemia in adulthood

Finally, we asked how early HFD challenges and the diet-dependent progenitor responses could affect adipose tissue cellularity and expansion in adulthood. To examine whether progenitors proliferating during early HFD could in principle contribute to adipose tissue composition in adulthood, we analyzed mice at 10 weeks of age which were transiently fed HFD with simultaneous EdU administration after weaning. 40% of Lin^−^CD29^+^CD34^+^Sca1^+^ progenitor cells were EdU label-retaining cells in mice with early HFD, indicating substantial contribution to the adult progenitor pool and thereby to the potential for future tissue remodeling (Figure S3A,B). In addition, early-proliferating progenitors substantially contributed to the formation of new adipocytes (approx. 20%, Figure S3C,D), with EdU^+^ adipocytes in early-HFD mice displaying smaller size compared to early-CD mice, despite no change in depot size (Figure S3E,F). We next tested whether the early HFD challenge could affect the response of adipose tissue to a secondary HFD challenge in adulthood and dissected the differential contribution of calorie and lipid surplus. To this end, mice were fed HFD, R-HFD, or CD for 3 weeks at weaning followed by CD up to adulthood, at which time all groups were re-challenged with HFD for 12 weeks (Figure 4A). Body weight was transiently increased after 3 weeks of early HFD feeding compared to CD and R-HFD but the difference did not persist until adulthood, reflecting the normalization of energy balance after the early challenge (Figure 4B). Upon re-challenge, early-HFD resulted in accelerated gain in body mass due to fat accumulation (Figure 4B,C and S4A). Remarkably, early-HFD affected fat distribution, since gWAT mass was significantly increased, in contrast to iWAT (Figure 4D). Mice fed R-HFD at weaning were indistinguishable from early CD-fed mice in terms of fat accumulation in adulthood (Figure 4A–D), implying that excess calories are responsible for the increased susceptibility to obesity. To determine whether early-HFD affected the growth mode of adipose tissue during the secondary expansion in adulthood we examined adipocyte size and number, the latter depending on progenitor function. gWAT mass positively correlated with adipocyte size in early-CD mice but not in early-HFD or R-HFD mice (Figure 4E). Conversely, gWAT mass in early-HFD and early-R-HFD mice showed a significant positive correlation with total adipocyte number, whereas this was not significant in early-CD mice (Figure 4F). These data suggest that the early transient feeding with HFD shifted the expansion mode of adipose tissue in response to a second HFD in adulthood from hypertrophic to hyperplastic growth independently of early calorie load in childhood and fat accumulation in adulthood.

Finally, we asked whether calorie and lipid excess during post-weaning growth impacted on systemic insulin sensitivity in adulthood and how this was associated with alterations in adipose tissue growth. Fasting serum insulin levels, as an early hallmark of the metabolic syndrome [33], were not significantly altered when mice fed early transient HFD or R-HFD reached adulthood (Figure S4B). Intriguingly though, a second HFD challenge in adulthood resulted in substantially increased insulin levels and the HOMA-IR indicator of insulin resistance in early-HFD mice and a strong trend of higher levels in early-R-HFD compared to early-CD mice (Figure 4G,H). Insulin tolerance was compromised mildly but significantly in both early-HFD and -R-HFD groups (Figure S4C). Insulin levels across all groups showed a strong positive correlation with gWAT mass (Figure 4I). Whereas insulin levels and the HOMA-IR were not associating with adipocyte size in gWAT, there was a significant positive correlation with adipocyte number, which was particularly evident in R-HFD mice (*r* = 0.71, *P* = 0.0096 for insulin) (Figure S4D-G). Taken together, these results demonstrate that hypercaloric HFD feeding during post-weaning growth can increase the susceptibility to abdominal obesity in adulthood and that early high dietary fat content can alter the expansion mode of adult adipose tissue in association with hyperinsulinemia independently of early excess calories.

## 4 Discussion

It is currently unclear how specific nutritional factors drive the proliferative growth of adipose tissue in childhood and adolescence, and, generally, little is known about the control of cell proliferation by dietary components [41]. Our results reveal that dietary lipids can promote immature progenitor cell proliferation in adipose tissue independently of excess caloric intake and fat accumulation. The cascade leading from excess nutrients to progenitor activation for adipogenesis has been proposed to be triggered by adipocytes undergoing excessive hypertrophy, mainly based on the fact that hypertrophy precedes the appearance of new adipocytes upon overfeeding [14], [42], [43]. Overloading of adipocytes is thought to stimulate local inflammation, which has been shown to play a role in the initial activation of adipogenesis [31], [32]. By modifying the calorie load during HFD feeding, we demonstrate that adipocyte hypertrophy and immune response-related gene expression depend on calorie excess and may contribute to progenitor proliferation. Importantly, we show that unexpectedly, high fat dietary content without caloric surplus promoted cell proliferation independently of these parameters in juvenile mice. It is noteworthy that R-HFD did not increase cell proliferation in 6-month-old mice. In agreement with recent reports, *ad libitum* HFD caused a trend of increased EdU incorporation, albeit at an overall low level [44]. These observations may reflect the reduced hyperplastic potential of adult adipose tissue [20], [45].

IGF-1 is a systemic growth-promoting factor, and its levels have been reported to be elevated in early-onset obesity [46], [47]. Our observation that systemic and local IGF-1 levels were increased in *ad libitum* HFD-fed, but not R-HFD-fed, mice reveals the importance of excess calories rather than lipids in the regulation of IGF-1 in obesity. IGF-1 enhanced the proliferation of isolated progenitor cells from young mice suggesting that elevated IGF-1 levels could mediate the effect of excess calories on cell proliferation. The effect of excess lipids on progenitor proliferation is likely to depend on locally increased fatty acid concentrations, given the abrogation of the proliferative response to HFD in *Cd36*-deficient mice, which is consistent with the reduced number of adipocytes observed in HFD-fed *Cd36*-null mice [48]. Indeed, we found that C16:1 palmitoleic acid, but not oleic, palmitic or polyunsaturated fatty acids, enhanced progenitor proliferation by increasing the sensitivity to IGF-1 in a cell-autonomous and *Cd36*-independent manner. Although the *ex vivo* proliferation assay cannot faithfully recapitulate the *in vivo* situation in terms of the concentration and delivery mode of fatty acids, it revealed a rather specific synergy between C16:1 and IGF-1. Combinatorial effects of fatty acids according to their relative abundance in the tissue of HFD-fed mice need to be considered in the future. Interestingly, adipose tissue palmitoleic acid content normally declines during childhood and two studies found a positive correlation between circulating free palmitoleic acid and abdominal obesity in children [38], [49], [50]. Palmitoleic acid has been demonstrated to act as an endocrine signal but further studies are required to determine the signaling mechanisms and the impact of excess dietary supply on early fat expansion [38].

Transient HFD feeding during post-weaning growth did not result in considerable increases in fat mass or fasting insulin levels in adulthood. Remarkably though, by employing a second HFD challenge in adulthood, we reveal increased susceptibility of early-HFD-fed mice to abdominal obesity and insulin resistance suggesting long-term nutritional programming of central features of the metabolic syndrome [1]. Increased intra-abdominal obesity by early HFD was dependent on the consumption of excess calories, highlighting the primary importance of this factor in childhood nutrition for long-term metabolic health. However, irrespective of the calorie load, high fat content in the early diet shifted the expansion mode of adipose tissue upon the second challenge in adulthood from adipocyte hypertrophy to hyperplasia. Thus, the early stimulation of progenitor proliferation by HFD and R-HFD, which was preferentially occurring in gWAT compared to iWAT, could contribute to increased potential for adipocyte formation in the adult intra-abdominal fat depot, including qualitative differences. Further studies are required to understand the contribution of early progenitor responses to depot-specific alterations in cellularity. Given the substantial sex-specific differences in adipose tissue biology, it will be very interesting to compare our findings to the dietary responses of male mice.

The association between intra-abdominal obesity and hyperinsulinemia observed in our model reflects the situation in humans, where visceral obesity coincides with systemic insulin resistance and represents a risk factor for type 2 diabetes and other chronic diseases [1], [3], [51]. Intriguingly though, insulin levels correlated with adipocyte number in gWAT rather than with adipocyte size. This is in contrast with the so-called expandability hypothesis, stating that expansion of adipose tissue by increasing the number of adipocytes is protective against insulin resistance through the increased capacity to safely store lipids while avoiding excessive adipocyte hypertrophy [2], [4], [20], [52]. On the other hand, some reports on human obesity reveal more complex relationships, with adipogenesis and smaller rather than larger adipocytes associated with insulin resistance [53], [54]. What distinguishes our mouse model is that dietary manipulation was performed during early growth of the mice. In particular, we suggest that qualitative changes are programmed by early dietary challenges in progenitors, their adipocyte progeny and their stroma which are likely to substantially influence systemic insulin sensitivity independently of effects on the quantity of adipocytes. Given the importance of lipids as components of milk for postnatal growth as well as the differential effects of fatty acid species on adipose tissue growth shown here and elsewhere [11], [55], it will be crucial to investigate the impact of dietary fat composition on the programming of tissue function and systemic metabolism.
