# Supplementary material for: Dietary calories and lipids synergistically shape adipose tissue cellularity during postnatal growth
Source: Mol Metab. 2019 Apr 5;24:139–48. doi: 10.1016/j.molmet.2019.03.012 (PMC6531874; doi:10.1016/j.molmet.2019.03.012)
Supplement: Multimedia component 1 [file mmc1.pdf]

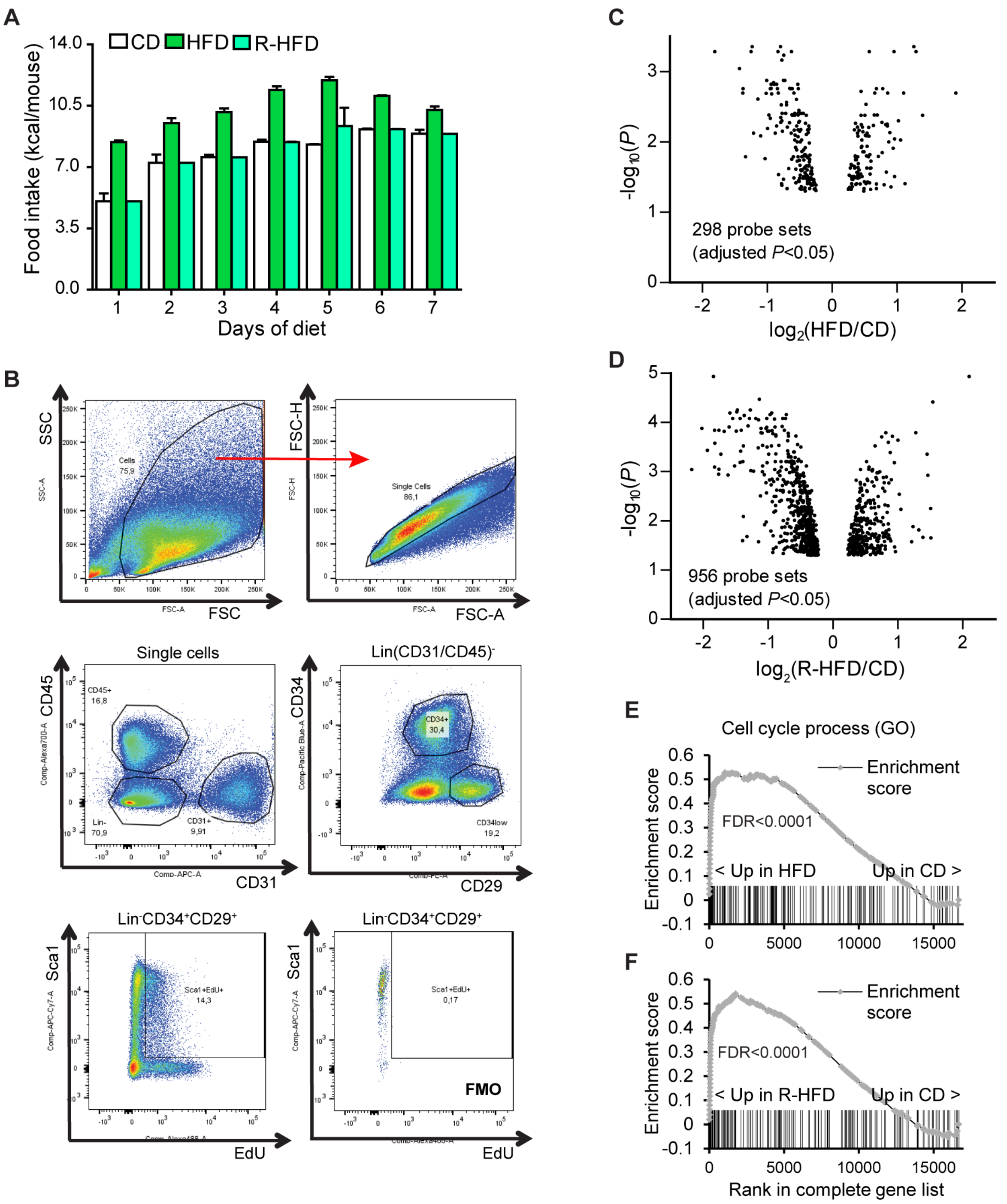

Figure S1. Dietary lipids accelerate adipose tissue progenitor proliferation independently of excess calories and adipocyte hypertrophy in juvenile mice.

(A) Daily food intake in 3-week-old mice fed control diet (CD), high-fat diet (HFD) or HFD calorically matched to CD (R-HFD) (n=10 mice).

(B) Representative flow cytometry sequential gating scheme. Single cell suspensions of dissociated adipose tissue were simultaneously stained for the indicated antigens. Plot titles indicate the population selected in the previous plots. Lower right panel is an FMO control for EdU.

(C, D) Volcano plots of differential RNA expression in gWAT from mice fed with the indicated diets for 1 week. The values for individual genes with *P*<0.05 are shown. *P* represents the *P*-value for the indicated comparison obtained by LIMMA with Benjamini-Hochberg adjustment.

The log_2-ratio of the indicated expression values is represented on the X-axis. (n=3/5/5 samples, each pooled from 2 mice).
(E, F) Enrichment plots for the indicated gene set [Gene Ontology (GO)] from GSEA on expression profiles from gWAT after one week of the indicated diets. Vertical bars indicate the rank of the individual genes of the indicated gene set in the ranking of the transcriptome by differential expression (n=3/5/5 samples, each pooled from 2 mice).
Data are presented as mean ± SD (A).
