# Supplementary material for: Dietary calories and lipids synergistically shape adipose tissue cellularity during postnatal growth
Source: Mol Metab. 2019 Apr 5;24:139–48. doi: 10.1016/j.molmet.2019.03.012 (PMC6531874; doi:10.1016/j.molmet.2019.03.012)
Supplement: Multimedia component 2 [file mmc2.pdf]

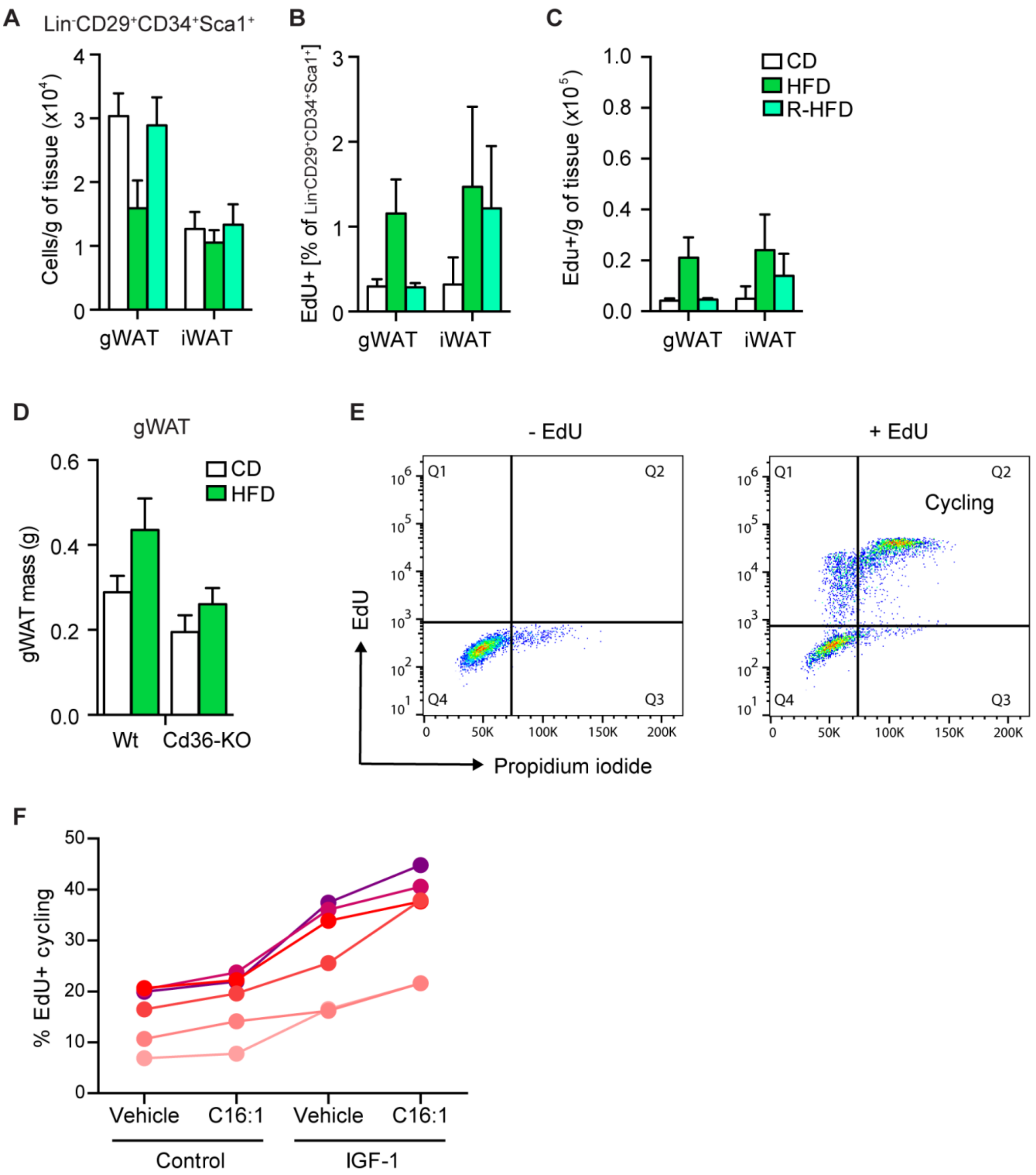


**Figure S2. Dietary lipid-dependent proliferation in adult mice and fatty acid-dependent progenitor proliferation in juvenile mice and ex vivo.**

(A) Number of Lin$^-$CD29$^+$CD34$^+$Sca1$^+$ cells per gram of tissue in 6-month-old mice after one week of CD, HFD and R-HFD (n=4 samples with cells pooled from 3 mice each).

(B, C) Frequency (B) and number per gram of tissue (C) of EdU$^+$ Lin$^-$CD29$^+$CD34$^+$Sca1$^+$ cells in 6-month-old mice after one week of CD, HFD and R-HFD with EdU-containing drinking water, determined by flow cytometry (n=5 mice for gWAT, n=4 mice for iWAT).

(D) gWAT mass after 7 days of CD or HFD in wildtype or Cd36-KO mice (n=4 mice).

(E) Flow cytometry of primary Lin$^-$Sca1$^+$ cells cultured with or without 5 µM EdU for 16 hours and stained for EdU (Alexa Fluor 647) and DNA content (propidium iodide). Cycling cells are identified as EdU$^+$ cells in S/G2/M (Q2).

(F) Frequency (%) of EdU$^+$ cycling (S/G2/M) Lin$^-$Sca1$^+$ cells in primary cultures from wild type gWAT following treatment with C16:1 and IGF-1 as indicated for 16 hours in the presence of EdU. Each series represents an independent biological replicate (n=6).

Data are presented as mean ± SEM (A-D). *$P$<0.05, **$P$<0.01, ***$P$<0.001 vs. CD and $^{\#}P$<0.05, $^{\#\#}P$<0.01, $^{\#\#\#}P$<0.001 vs. HFD (One-way ANOVA, posthoc Tukey) (A-C). Two-way ANOVA with Tukey posthoc tests (D). Representative plots (E). Biological replicates (F).
