# Supplementary material for: Dietary calories and lipids synergistically shape adipose tissue cellularity during postnatal growth
Source: Mol Metab. 2019 Apr 5;24:139–48. doi: 10.1016/j.molmet.2019.03.012 (PMC6531874; doi:10.1016/j.molmet.2019.03.012)
Supplement: Multimedia component 3 [file mmc3.pdf]

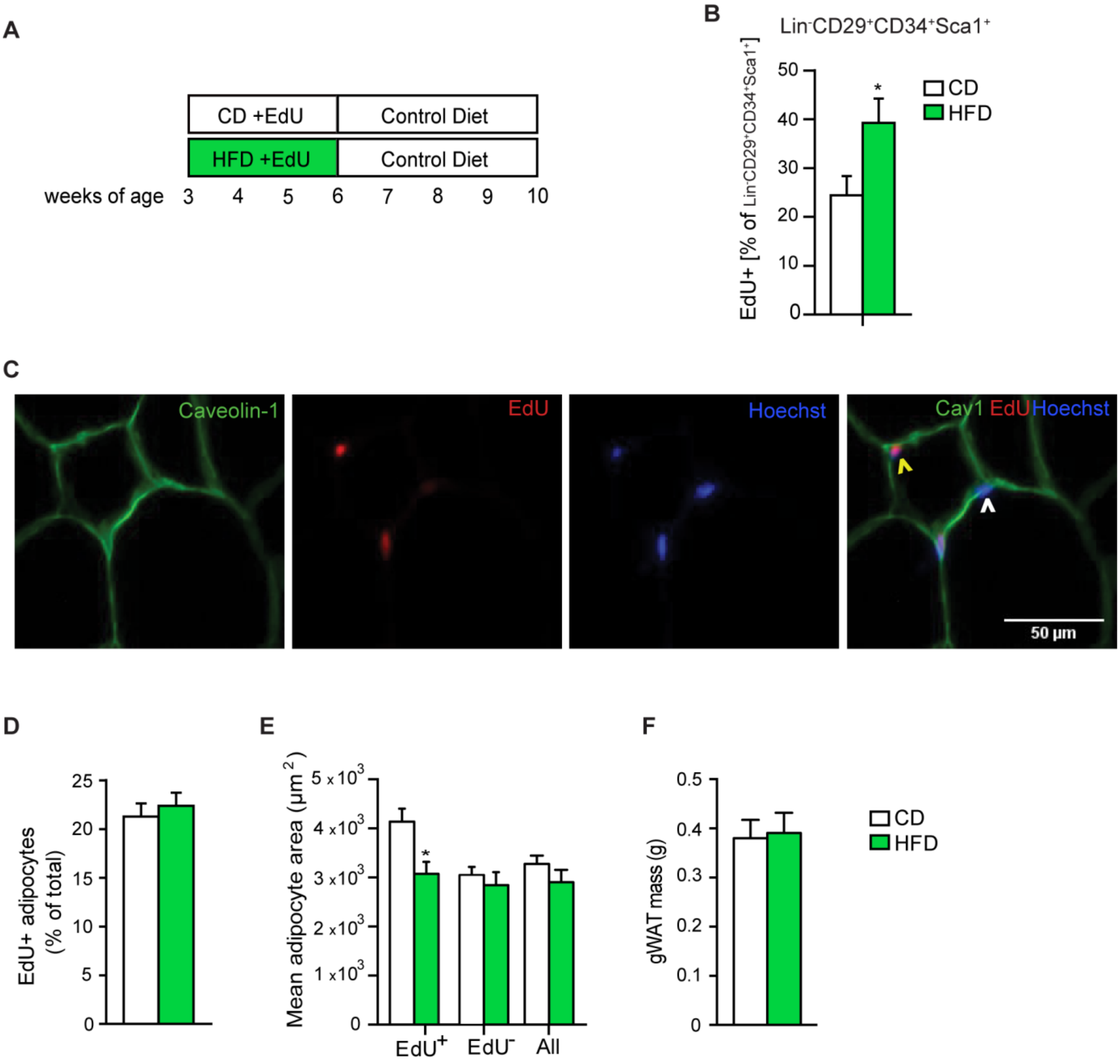

Figure S3. Contribution of progenitors proliferating during transient post-weaning HFD to the adult adipose tissue composition.

(A) Experimental design for Figure 3. 3-week-old mice were fed CD or HFD for 3 weeks with EdU-containing drinking water, followed by control diet up to 10 weeks of age (woa).

(B) Frequency of EdU^+ $Lin^-CD29^+CD34^+Sca1^+$ cells in gWAT of mice in (A) at 10 woa, determined by flow cytometry (n=8 mice).

(C) Representative images in the indicated fluorescence channels of gWAT section stained for Caveolin-1 (Cav1), EdU and Hoechst. Yellow arrow indicates nucleus within the adipocyte membrane, white arrow indicates nucleus of adjacent non-adipocyte cell. Scale bar: 50 µm.

(D) Frequency of EdU^+ adipocytes in gWAT, determined by quantitative microscopy of histological sections as in (C) (n=8 mice).

(E) Mean area of adipocytes in gWAT, determined by quantitative microscopy of histological sections as in (C) (n=8 mice).

(F) gWAT mass (n=8 mice).

Data are presented as mean ± SEM. *P<0.05 (*t*-test).
