# Supplementary material for: Dietary calories and lipids synergistically shape adipose tissue cellularity during postnatal growth
Source: Mol Metab. 2019 Apr 5;24:139–48. doi: 10.1016/j.molmet.2019.03.012 (PMC6531874; doi:10.1016/j.molmet.2019.03.012)
Supplement: Multimedia component 4 [file mmc4.pdf]

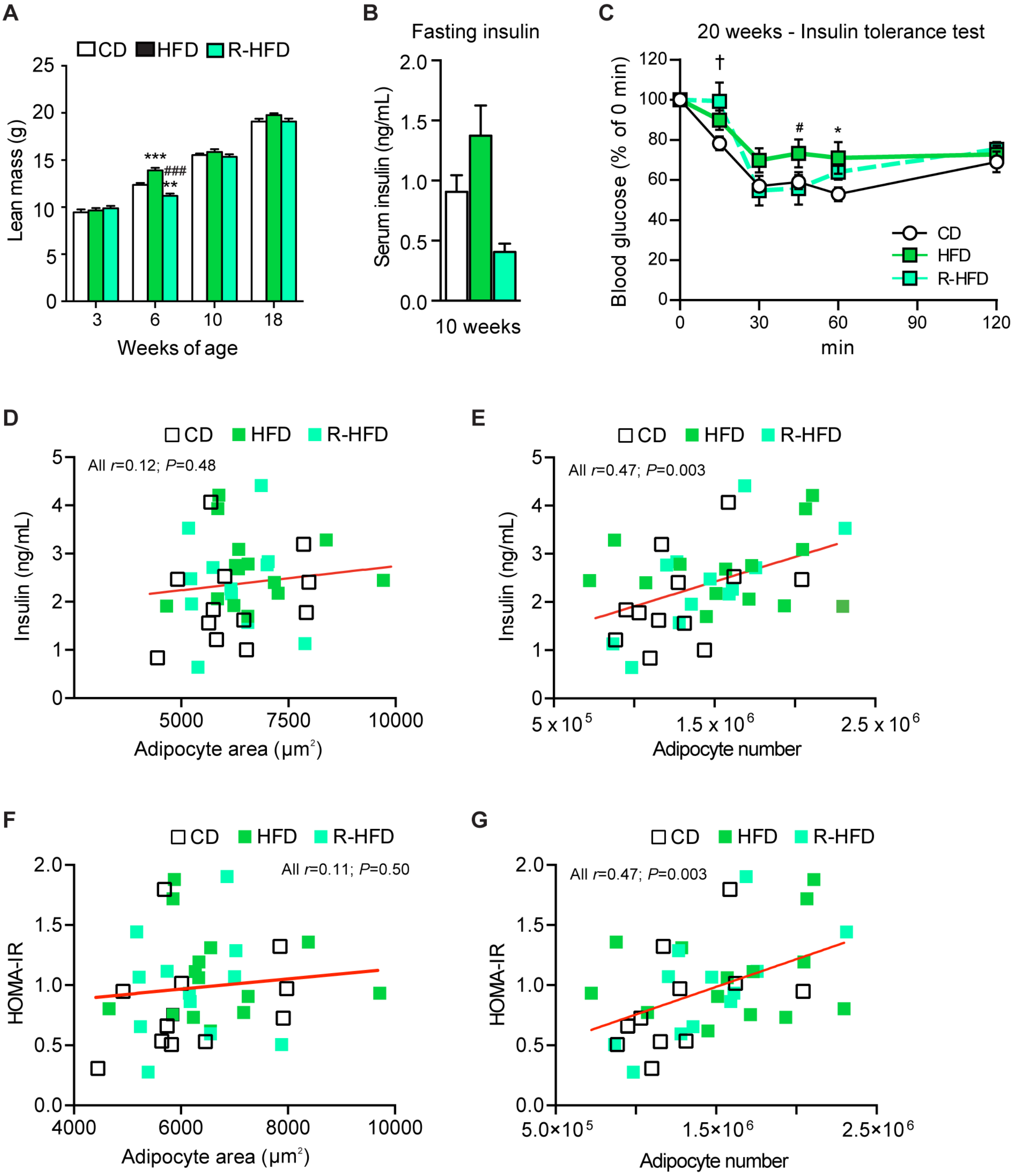

Figure S4. Excess calories and fat during post-weaning growth differentially alter the susceptibility to abdominal hyperplastic obesity and hyperinsulinemia in adulthood.

(A) Body lean mass, determined by NRM quantitation (n=14 mice). 3-week-old mice were fed CD, HFD or R-HFD for 3 weeks, followed by control diet for 4 weeks (all 3 groups) and subsequently HFD for 12 weeks (all 3 groups).

(B) Fasting serum insulin, determined by ELISA in mice shown in (A) at 10 weeks of age (woa) (n=14/17/6 mice).

(C) Blood glucose during insulin tolerance test in mice shown in (A) at 20 woa (n=8 mice).

(D, E) Pearson correlation analysis of fasting serum insulin (ELISA) and mean adipocyte area (D) or total adipocyte number (E) in gWAT at 22 woa (n=12-14 mice).

(F, G) Pearson correlation analysis of the HOMA-IR and mean adipocyte area (F) or total adipocyte number (G) in gWAT at 22 woa (n=12-14 mice).

Data are presented as mean ± SEM (A-C) or individual mice (D-G). (A,B) **$P<0.001$, ***$P<0.001$ vs CD and $^{\#\#\#}P<0.001$ vs HFD (One-way ANOVA with Tukey posttests); (C) $^{\dagger}P<0.05$ R-HFD vs CD, *$P<0.05$ HFD vs CD, $^{\#}P<0.05$ HFD vs R-HFD (2-way ANOVA with Tukey posttests).
