# Supplementary material for: Dietary calories and lipids synergistically shape adipose tissue cellularity during postnatal growth
Source: Mol Metab. 2019 Apr 5;24:139–48. doi: 10.1016/j.molmet.2019.03.012 (PMC6531874; doi:10.1016/j.molmet.2019.03.012)
Supplement: Multimedia component 5 [file mmc5.pdf]

**Table S1. Enriched gene sets upregulated upon 7 days of HFD[1].**

| Gene set name | Enrichment score | FDR q-value |
|---|---|---|
| SPINDLE | 0.78 | <0.0001 |
| MITOSIS | 0.66 | <0.0001 |
| M_PHASE_OF_MITOTIC_CELL_CYCLE | 0.66 | <0.0001 |
| M_PHASE | 0.61 | <0.0001 |
| MITOTIC_CELL_CYCLE | 0.56 | <0.0001 |
| CELL_CYCLE_PROCESS | 0.53 | <0.0001 |
| MICROTUBULE_CYTOSKELETON | 0.53 | <0.0001 |
| CHROMOSOMEPERICENTRIC_REGION | 0.72 | <0.0001 |
| CELL_CYCLE_PHASE | 0.52 | <0.0001 |
| CHROMOSOME_SEGREGATION | 0.68 | 0.0001 |
| SPINDLE_MICROTUBULE | 0.80 | 0.0003 |
| DNA_PACKAGING | 0.66 | 0.0006 |
| REGULATION_OF_MITOSIS | 0.64 | 0.0007 |
| CHROMOSOME | 0.52 | 0.0011 |
| CELL_CYCLE_GO_0007049 | 0.45 | 0.0012 |
| KINESIN_COMPLEX | 0.80 | 0.0013 |
| SISTER_CHROMATID_SEGREGATION | 0.76 | 0.0013 |
| SPINDLE_ORGANIZATION_AND_BIOGENESIS | 0.85 | 0.0017 |
| MICROTUBULE_MOTOR_ACTIVITY | 0.76 | 0.0018 |
| CELLULAR_DEFENSE_RESPONSE | 0.59 | 0.0018 |
| SPINDLE_POLE | 0.74 | 0.0018 |
| CHROMOSOMAL_PART | 0.53 | 0.0019 |
| MICROTUBULE_CYTOSKELETON_ORGANIZATION_AND_BIOGENESIS | 0.63 | 0.0021 |
| MITOTIC_SISTER_CHROMATID_SEGREGATION | 0.76 | 0.0021 |
| CYTOSKELETAL_PART | 0.45 | 0.0022 |
| CONDENSED_CHROMOSOME | 0.63 | 0.0026 |
| KINETOCHORE | 0.68 | 0.0046 |
| MICROTUBULE_ORGANIZING_CENTER | 0.53 | 0.0048 |
| MOTOR_ACTIVITY | 0.64 | 0.0048 |
| CHROMOSOME_CONDENSATION | 0.83 | 0.0048 |
| CENTROSOME | 0.55 | 0.0051 |
| BRAIN_DEVELOPMENT | 0.55 | 0.0074 |
| CYTOSKELETON_ORGANIZATION_AND_BIOGENESIS | 0.44 | 0.0084 |
| ACTIN_CYTOSKELETON_ORGANIZATION_AND_BIOGENESIS | 0.49 | 0.0085 |
| CELL_CYCLE_CHECKPOINT_GO_0000075 | 0.55 | 0.0112 |
| ESTABLISHMENT_OF_ORGANELLE_LOCALIZATION | 0.71 | 0.0113 |
| MICROTUBULE | 0.60 | 0.0133 |
| CARBOHYDRATE_BINDING | 0.51 | 0.0171 |
| ACTIN_POLYMERIZATION_AND_OR_DEPOLYMERIZATION | 0.65 | 0.0186 |
| CYTOSKELETON | 0.39 | 0.0277 |
| POSITIVE_REGULATION_OF_CELL_CYCLE | 0.68 | 0.0319 |
| MITOTIC_CELL_CYCLE_CHECKPOINT | 0.64 | 0.0323 |
| POSITIVE_REGULATION_OF_IMMUNE_RESPONSE | 0.59 | 0.0325 |
| PHOSPHOLIPASE_C_ACTIVATION | 0.72 | 0.0327 |

| | | |
|---|---|---|
| TISSUE_DEVELOPMENT | 0.44 | 0.0327 |
| IMMUNE_SYSTEM_PROCESS | 0.40 | 0.0330 |
| IMMUNE_RESPONSE | 0.41 | 0.0364 |
| LEADING_EDGE | 0.53 | 0.0370 |
| POSITIVE_REGULATION_OF_IMMUNE_SYSTEM_PROCESS | 0.52 | 0.0371 |
| CELL_DIVISION | 0.65 | 0.0395 |
| ACTIN_FILAMENT_BASED_PROCESS | 0.44 | 0.0416 |
| MESODERM_DEVELOPMENT | 0.62 | 0.0444 |
| MICROTUBULE_BASED_PROCESS | 0.46 | 0.0544 |
| CELL_PROJECTION_BIOGENESIS | 0.59 | 0.0546 |
| CYTOKINESIS | 0.65 | 0.0557 |
| CELL_MIGRATION | 0.44 | 0.0561 |
| CHROMOSOME_ORGANIZATION_AND_BIOGENESIS | 0.43 | 0.0590 |
| SUGAR_BINDING | 0.56 | 0.0594 |
| PROTEIN_HETERODIMERIZATION_ACTIVITY | 0.46 | 0.0646 |
| PHOSPHOLIPASE_C_ACTIVITY | 0.64 | 0.0672 |
| SULFURIC_ESTER_HYDROLASE_ACTIVITY | 0.69 | 0.0768 |
| COLLAGEN_BINDING | 0.68 | 0.0796 |
| PROTEIN_DIMERIZATION_ACTIVITY | 0.39 | 0.0888 |
| ENDOPLASMIC_RETICULUM_LUMEN | 0.68 | 0.0893 |
| REGULATION_OF_IMMUNE_RESPONSE | 0.54 | 0.0914 |
| RUFFLE | 0.55 | 0.0919 |
| BASOLATERAL_PLASMA_MEMBRANE | 0.51 | 0.0922 |
| POSITIVE_REGULATION_OF_MULTICELLULAR_ORGANISMAL_PROCESS | 0.47 | 0.0931 |
| ACTIN_FILAMENT_POLYMERIZATION | 0.67 | 0.0932 |
| LAMELLIPODIUM | 0.57 | 0.0954 |

[1] Gene Set Enrichment Analysis (GSEA) on microarray RNA expression profiles from gWAT of 3-week-old mice fed high-fat diet (HFD) or control diet (CD) for one week (n= 3 (CD), 5 (HFD) samples, each pooled from 2 mice). GSEA comparing HFD vs. CD was performed with the Gene Ontology (GO) gene set collection. Gene sets with False Discovery Rate (FDR) q-values lower than 10% are shown.
