# Supplementary material for: Dietary calories and lipids synergistically shape adipose tissue cellularity during postnatal growth
Source: Mol Metab. 2019 Apr 5;24:139–48. doi: 10.1016/j.molmet.2019.03.012 (PMC6531874; doi:10.1016/j.molmet.2019.03.012)
Supplement: Multimedia component 6 [file mmc6.pdf]

Table S2. Enriched gene sets upregulated upon 7 days of R-HFD[1].

Gene set name	Enrichment score	FDR q-value
SPINDLE	0.78	<0.0001
M_PHASE	0.62	<0.0001
MITOSIS	0.64	<0.0001
M_PHASE_OF_MITOTIC_CELL_CYCLE	0.65	<0.0001
MITOTIC_CELL_CYCLE	0.59	<0.0001
CHROMOSOMEPERICENTRIC_REGION	0.79	<0.0001
CELL_CYCLE_PROCESS	0.54	<0.0001
CONTRACTILE_FIBER	0.77	<0.0001
CHROMOSOME_SEGREGATION	0.73	<0.0001
STRUCTURAL_CONSTITUENT_OF_MUSCLE	0.72	<0.0001
CELL_CYCLE_PHASE	0.53	<0.0001
SPINDLE_MICROTUBULE	0.83	0.0001
KINETOCHORE	0.74	0.0001
MYOFIBRIL	0.79	0.0001
SARCOMERE	0.85	0.0001
CONTRACTILE_FIBER_PART	0.76	0.0002
CHROMOSOME	0.54	0.0002
CHROMOSOMAL_PART	0.55	0.0002
MICROTUBULE	0.69	0.0003
MICROTUBULE_CYTOSKELETON	0.51	0.0006
MICROTUBULE_MOTOR_ACTIVITY	0.79	0.0007
ESTABLISHMENT_OF_ORGANELLE_LOCALIZATION	0.80	0.0007
CELL_CYCLE_GO_0007049	0.46	0.0007
CHROMOSOME_CONDENSATION	0.91	0.0007
KINESIN_COMPLEX	0.83	0.0009
SISTER_CHROMATID_SEGREGATION	0.79	0.0010
MITOTIC_SISTER_CHROMATID_SEGREGATION	0.79	0.0010
CONDENSED_CHROMOSOME	0.65	0.0010
BRAIN_DEVELOPMENT	0.60	0.0012
ORGANELLE_LOCALIZATION	0.69	0.0013
CYTOSKELETAL_PART	0.46	0.0019
SPINDLE_POLE	0.72	0.0030
CHROMOSOME_ORGANIZATION_AND_BIOGENESIS	0.49	0.0051
AXON_GUIDANCE	0.68	0.0051
DNA_PACKAGING	0.62	0.0051
SPINDLE_ORGANIZATION_AND_BIOGENESIS	0.83	0.0067
REGULATION_OF_MUSCLE_CONTRACTION	0.71	0.0068
MOTOR_ACTIVITY	0.62	0.0074
CYTOKINESIS	0.69	0.0121
CELL_DIVISION	0.68	0.0122
CELL_MATRIX_JUNCTION	0.68	0.0128
CENTROSOME	0.52	0.0163
REGULATION_OF_MITOSIS	0.57	0.0187
MORPHOGENESIS_OF_AN_EPITHELIUM	0.69	0.0188

CYTOSKELETON	0.39	0.0214
MICROTUBULE_CYTOSKELETON_ORGANIZATION_AND_BIOGENESIS	0.57	0.0220
BASOLATERAL_PLASMA_MEMBRANE	0.55	0.0307
HEMATOPOIETIN_INTERFERON_CLASSD200_DOMAIN_CYTOKINE_RECEPTOR_BINDING	0.65	0.0313
NEGATIVE_REGULATION_OF_CELL_DIFFERENTIATION	0.57	0.0437
MICROTUBULE_ORGANIZING_CENTER	0.49	0.0465
CYTOSKELETON_ORGANIZATION_AND_BIOGENESIS	0.40	0.0498
CELL_SUBSTRATE_ADHERENS_JUNCTION	0.65	0.0504
MICROTUBULE_BASED_PROCESS	0.46	0.0588
SOLUTE_SODIUM_SYMPORTER_ACTIVITY	0.74	0.0596
STRIATED_MUSCLE_CONTRACTION_GO_0006941	0.70	0.0651
NUCLEAR_UBIQUITIN_LIGASE_COMPLEX	0.73	0.0673
PROTEIN_HOMODIMERIZATION_ACTIVITY	0.42	0.0711
MYELOID_CELL_DIFFERENTIATION	0.53	0.0718
SMOOTH_MUSCLE_CONTRACTION_GO_0006939	0.70	0.0743
CYTOSKELETAL_PROTEIN_BINDING	0.40	0.0833
ENDOTHELIAL_CELL_PROLIFERATION	0.71	0.0837
MYOBLAST_DIFFERENTIATION	0.62	0.0849

[1] Gene Set Enrichment Analysis (GSEA) on microarray RNA expression profiles from gWAT of 3-week-old mice fed calorically matched high-fat diet (R-HFD) or control diet (CD) for one week (n= 3 (CD), 5 (R-HFD) samples, each pooled from 2 mice). GSEA comparing HFD vs. CD was performed with the Gene Ontology (GO) gene set collection. Gene sets with False Discovery Rate (FDR) q-values lower than 10% are shown.
